# Prosopalgia—Tic Douloureux

**Published:** 1858-01

**Authors:** James B. Dixon


### ARTICLE XVI.

*Prosopalgia—Tic Douloureux.*—By JAMES B. DIXON, D. D. S.

FACEACHE, or facial neuralgia, is perhaps one of the most painful affections to which the body is liable, both from the violence of the paroxysms, and their frequent periodic occurrence. The pains are of a plunging, darting, digging, twitching character, and occur as if the face under the eye, in front of the ear, over the forehead, and down the neck, and to the root of the tongue, were suddenly struck; and the remission to ease is as sudden, leaving the part as free from pain as if the paroxysm had never occurred, until within perhaps a few minutes before the same tragic suffering is to be again endured, till replaced by sudden tranquillity. These fits of torture are of longer or shorter duration, sometimes finishing in an hour, at other times lasting weeks and months, with only occasional remissions.

The cause of this affection is not satisfactorily clear. Its exact origin is as yet obscnre. Sometimes it is attributed to a diseased growth of bone in different parts of the head and face especially about the canals through which the nerves pass; at other times it is attributed to irritation excited by foreign bodies acting upon some of the dental branches; it is also attributed to a constitutional cause, such as derangement of the general health; it is also supposed to depend upon an inflammatory state of some portion of the nervous system; and again it is attributed to some affection of the teeth, and morbid condition of the gums and mouth. Damp and cold are specified as exciting causes—damp combined with malaria such as produces ague, exposure to currents of cold air, especially if the body is overheated; debility of constitution, anxiety of mind, temporary stomach disorders, such as superabundant acid, are all put down as originators of this painful disease. It is therefore perfectly clear that that it must spring from a variety of causes, or else, that information as to its origin is indefinite and unsatisfactory, and its actual cause is obscure. The observations of Dr. Togg, in that valuable paper on "Odontalgia," as quoted in your Journal of July, do not add more positive information. He refers to a fibro-cartilaginous condition of the gasserian ganglion and its enlargement, but dissection does not confirm this as a general cause; hence its existence was only an additional condition of disease.

In neuralgia there does not appear to be constant lesion of the solids, nor evident alteration of the fluids, and hence the disease is supposed to be one of nervous influence; and as dissection affords no diagnostic results, not even so much as a diseased condition of the affected nerve, it is highly probable that this opinion is correct, and that it results from electrical influences, like the flashes of lightning disturbing the equilibrium of nature. Whether nervous fluid be identical to electric fluid, or whether it be subject only to kindred currents, may be matters of speculation; but it is highly probable that the paroxysms of tic are disturbances in the ordinary current of nervous fluid.

In the treatment of this dreadful affection the dentist is frequently consulted, and perhaps such cases lie more rationally within the province of his practice. It cannot however be inconsistent, under any circumstances, to afford relief in pain, for it is but the development of the social principle to render assistance in cases of necessity.

The remedies prescribed are various and manifold. Change of climate, dependent upon circumstances, such as the situation and temperature where the disease commenced; change either to a warm dry air, or *vice versa*, to a moist, humid, warm atmosphere. A course of medicines for indigestion—after that, tonics—such as quinine, carbonate of iron, carbonate of soda, &c., blisters behind the ear, and at the back of the neck. The late Sir Charles Bell recommended a drastic aperient pill, composed of croton oil and compound extract of colocynth, and an antispasmodic, the compound galbanum pill. A piece of sponge, or flannel, dipped in boiling water, and applied, has also been advised. Opium internally and externally administered. Chloroform applied over the affected part. Galvanism, electricity, and the surgical operation of the division of the nerve, are also recommended.

Such is the catalogue of remedies, as directed by medical writers, for the removal of this terrific disease. Occasionally, one or other of them succeeds, but unfortunately, in too many instances, no satisfactory result follows. The whole string of remedies sometimes fails even in affording only temporary relief. In a special case, which came under my own treatment and observation, I had ample opportunity of testing the value of the accredited remedies, and I must confess the result was highly dissatisfactory. My father, the Rev. M. C. D——, ætat 71, became a sufferer under an attack of tic about two years ago. He took quinine, carbonate of iron, aperients, arsenic, &c., without relief. I consulted with an eminent M. D. and he prescribed bebearine, but with no better success. After a three months' course, the disease disappeared, and the conclusion to which I came was that the medicines were valueless in the attack. Six months afterwards, the paroxysms returned, and continued in defiance of remedies for about a month. He was at that time in Lowestoft, and had the advice of two medical practitioners, who prescribed for him. Towards the end of last year, the tic again recurred, and continued for about two months. He was then in Manchester, and was under the advice of an eminent surgeon. In May of this year, whilst at my house, the same affliction returned. He took, under my direction, quinine, carbonate of iron. I applied a chloroform liniment to the face, and was astounded at the paroxysm actually recurring during the application. Tincture of opium was also rubbed on the painful part, and applied to the ear on cotton wool. Supposing the strokes to originate in the mouth, from some carious roots, I advised their removal, and took out five in one day, and three the day following, but no relief was obtained. I then applied to a friend of mine, who professed great faith in homœopathy, and he advised me to give one globule of belladonna, and of bryonia, alternately, every half-hour, and the cure was effected within twenty-four hours. This was indeed, a highly satisfactory result, and in the hope of obtaining some data for the origination of this complaint, I investigated the specific action of these drugs, with the following results. Belladonna (deadly nightshade.) From experiments made with this drug upon animals of different species, we have various results. Goats, rabbits, &c., which live on vegetable diet, are almost innocuous to the action of belladonna, and can eat it with impunity; whilst carnivorous animals, such as dogs, cats, &c., are more sensitive to its action; and the conclusion derived from this fact is this drug is more pernicious to carnivorous, than to herbivorous animals. The therapeutic problem derived from this fact is, that the specific action of belladonna is in exact ratio to the development and functional activity of the brain upon which this poison produces direct effect, and man of all animals is most susceptible of its action; and experiment has established this further fact, that those are most powerfully affected, whose cerebral faculties are most liable to become irritated by particular causes, or whose brains have the most complete development, for idiots appear to be protected from the deleterious action of belladonna, and can eat it with the same impunity as animals of an inferior order. In applying these observations to neuralgia, we should infer that the disease was connected with cerebral excitement, whilst the *location* of the development, was the result of specific action, or local irritation, so that it would lead to the same conclusion we have already premised as the cause of facial neuralgia, that of nervous irritation, and disturbance in the current of nervous fluid. A strong infusion of coffee, is an antidote for belladonna, and consequently not suitable for persons afflicted with the tic.

Bryonia (white bryony,) is found to act upon the muscles, and especially upon fibrous tissues, which would lead to the conclusion, that the current of nervous fluid was impeded, or disturbed by muscular or fibrous contraction where the paroxysms occur.

I have tried the tincture of belladonna, and tincture of bryonia, in three other cases with the same marked success, the relief being in every case almost instantaneous.

*High Street, Hanley, Staffordshire.* *Ib.*

